# Fast, Reliable, and Simple Point-of-Care-like Adaptation of RT-qPCR for the Detection of SARS-CoV-2 for Use in Hospital Emergency Departments

**DOI:** 10.3390/v13122413

**Published:** 2021-12-02

**Authors:** Martina Pavletić, Marija Mazor, Mate Lerga, Tatjana Mileta, Jelena Železnjak, Tina Ružić, Sanda Ravlić, Dora Palčevski, Jelena Kirinčić, Silvestar Mežnarić, Ana Mišković, Maja Materljan, Alan Šustić, Berislav Lisnić, Vanda Juranić Lisnić

**Affiliations:** 1 Emergency Department, Clinical Hospital Center Rijeka, 51000 Rijeka, Croatia; martinapavletic1@gmail.com (M.P.); mate.lerga@gmail.com (M.L.); mileta_tatjana@yahoo.com (T.M.); dora.palcevski@gmail.com (D.P.); jelenakirincic@net.hr (J.K.); silvestar.meznaric@student.uniri.hr (S.M.); amiskovic@gmail.com (A.M.); unakokic@gmail.com (M.M.); 2 Department of Anesthesiology, Reanimatology, Intensive Care and Emergency Medicine, Faculty of Medicine, University of Rijeka, 51000 Rijeka, Croatia; alan.sustic@uniri.hr; 3 Center for Proteomics, Faculty of Medicine, University of Rijeka, 51000 Rijeka, Croatia; marija.mazor@uniri.hr (M.M.); jelena.zeleznjak@uniri.hr (J.Ž.); tjenus@uniri.hr (T.R.); berislav.lisnic@uniri.hr (B.L.); 4 Centre for Research and Knowledge Transfer in Biotechnology, University of Zagreb, 10000 Zagreb, Croatia; sravlic@unizg.hr

**Keywords:** SARS-CoV-2, qPCR, direct qPCR, dqPCR, point-of-care PCR, emergency department, molecular diagnostics, COVID-19

## Abstract

During COVID-19 pandemics, the availability of testing has often been a limiting factor during patient admissions into the hospital. To circumvent this problem, we adapted an existing diagnostic assay, Seegene Allplex SARS-CoV-2, into a point-of-care-style direct qPCR (POC dqPCR) assay and implemented it in the Emergency Department of Clinical Hospital Center Rijeka, Croatia. In a 4-month analysis, we tested over 10,000 patients and demonstrated that POC-dqPCR is robust and reliable and can be successfully implemented in emergency departments and similar near-patient settings and can be performed by medical personnel with little prior experience in qPCR.

## 1. Introduction

Accurate and fast diagnosis is a cornerstone of medicine in general and is especially important during pandemics. As the incidence of infection increases, a high burden is placed on the hospitals to quickly and accurately identify infectious patients and personnel to prevent intra-hospital transmission of a disease that could be fatal to sensitive patients and jeopardize the availability and quality of healthcare. The ongoing COVID-19 pandemics provided ample examples of these facts [1,2].

Quantitative PCR (qPCR) based diagnostic procedures, and associated primers and probes, were developed within a month of the Wuhan outbreak and have remained the gold standard for SARS-CoV-2 diagnosis due to their high sensitivity, specificity, and suitability for various types of samples [3,4,5]. However, qPCR-testing for SARS-CoV2 requires highly trained personnel and dedicated BSL-2 facilities suited for RNA isolation and diagnostic qPCR. Although most hospitals possess specialized laboratories capable of performing SARS-CoV-2 detection by qPCR, the collection of samples at various sites within hospitals and their transport to dedicated laboratories increases the time from sample acquisition to result.

During periods of a high incidence of SARS-CoV-2 infection (pandemic waves), the waiting time from sample collection to results becomes a significant burden to hospitals, especially emergency departments (EDs) that manage new patients and perform emergency procedures. Therefore, EDs are potential hotspots for intrahospital transmission of SARS-CoV-2, especially when the infection rates in the local population are high and asymptomatic carriers may still transmit the virus [6,7,8].

In Croatia, guidelines mandate that all patients with an unknown COVID-19 infection status that require hospital admission must be separated in isolation rooms from COVID-19-negative patients to prevent the spread of the virus. Consequently, due to the limited number of isolation rooms, especially in intensive care units, the speed and testing capacity become limiting factors during pandemic waves. [9]. Regrettably, even though fast and simplified tests adapted for use by non-expert personnel (known as point-of-care tests (POCT) are not a new concept and are available on the market, their cost per patient is significantly higher than for non-POCT variants. In addition, POCT tests usually suffer from lower throughput (processivity of only several patients per run) and are difficult to obtain due to high demand [3]. To overcome these hurdles and ensure the optimal functioning of the ED and the hospital in general, we optimized and tested an existing commercially available and validated test, Allplex SARS-CoV-2 Assay (Seegene), for use as a POCT-style assay so that it could be performed by clinical doctors and technicians with little prior experience in qPCR within the CHC Rijeka’s ED and other near-patient settings. We then implemented an on-site SARS-CoV-2 diagnostics laboratory within the ED of Clinical Hospital Center Rijeka (CHC Rijeka) and analyzed its work during a 4-month period.

qPCR-based detection of SARS-CoV-2 consists of several steps: (1) sampling (mostly nasopharyngeal and oropharyngeal swabs), (2) storage of patient samples and transfer to a dedicated diagnostic laboratory, (3) RNA isolation, (4) reverse transcription, and (5) detection of SARS-CoV-2 genome regions by qPCR. Of those, steps 2 and 3 proved to be the most time-consuming in CHC Rijeka, resulting in up to 36 h of waiting time from sampling to results. Such an extended timespan, in turn, culminated in an increased burden on CHC Rijeka’s capacity, and similar issues have been reported for other EDs elsewhere during the COVID-19 pandemics [10]. Performing a qPCR-based diagnostic test within the ED can effectively circumvent step 2 of the standard qPCR procedure. When performed by non-expert personnel, RNA isolation (step 3) can be a source of false-positive and false-negative results due to improper handling (cross-contamination of samples, the introduction of RNases, etc.). We therefore decided to optimize a test procedure for direct qPCR (dqPCR) detection without the RNA isolation step, which had already been demonstrated as possible with several different commercially available qPCR reagents [11,12,13,14,15,16]. Seegene’s Allplex SARS-CoV-2 Assay was selected as optimal for our POC-style adaptation, as the kit comes with SARS-CoV-2 Viewer, software for automated data interpretation, and thus does not require prior experience in qPCR result read-outs. It has also recently been demonstrated as highly reliable and sensitive [17].

We evaluated the feasibility of implementing optimized and simplified qPCR procedures within the ED and similar near-patient settings for use by medical doctors and technicians with little or no prior experience with qPCR. We demonstrated that such POC-style direct qPCR detection of SARS-CoV-2 did not result in diminished sensitivity and specificity compared to standard procedures involving RNA isolation. Furthermore, the increase in the processivity and speed of testing resulted in a decreased burden on CHC Rijeka, which, prior to the implementation of this test, had to place all COVID-19 suspect patients requiring hospital admission and stays into separate rooms until they were tested. Increased processivity and testing speed also allowed us to regularly implement routine testing of all personnel working in COVID departments or the ED (every 48–72 h). Hospital workers are not only individuals at increased risk for COVID-19, especially if they work in COVID-designated departments or EDs, but can also be important factors in intrahospital transmissions of the disease [18,19,20,21,22].

## 2. Materials and Methods

### 2.1. Study Participants and Sample Collection

For the initial dqPCR vs. stndqPCR method comparison, we included four groups of study participants: (1) patients arriving at the ED with indications for SARS-CoV-2 testing (criteria: symptoms or close contact with known COVID-19 patients), (2) CHC Rijeka personnel working in the ED, (3) COVID-19 patients hospitalized in one of the respiratory centers (CRC patients), and (4) COVID-19 patients with confirmed infection 2 or more weeks prior to the test (CRI patients). After obtaining informed consent, patients were swabbed twice consecutively using synthetic swabs (Copan). One oropharyngeal and one nasopharyngeal swab were placed into 2 mL of Universal transport medium (UTM) (Copan) to obtain a UTM sample. Another oropharyngeal swab was placed into 2 mL of molecular grade (MG), RNAse, DNA, and DNAse free water within the RNA and RNAse-free 15 mL conical tube (Falcon) to obtain an MG sample. All types of samples were stored at +4 °C for a maximum of 5 h until processing. The swabs were shipped on ice packs in thermo-bags or Styrofoam boxes if transport to another location was needed. If samples could not be further processed within 5 h, they were stored at −80 °C. For all other tests, both oropharyngeal and nasopharyngeal swabs from each patient were placed in DNA, RNAse, and RNA-free 15 mL conical tubes filled with 2 mL molecular grade (MG) water and processed within 3 h from sample collection. MG water was obtained by purifying demineralized water on an OmniaPure UV/UF (StakPure Gmbh, Niederahr, Germany) water purifier. For analyses of pooled samples, up to 5 oropharyngeal swabs were placed in the same sample tube containing 2 mL of MG water and then stored and processed as the single-swab samples in MG water.

### 2.2. Sample Processing, RNA Isolation and RT-qPCR

**Figure 1:**
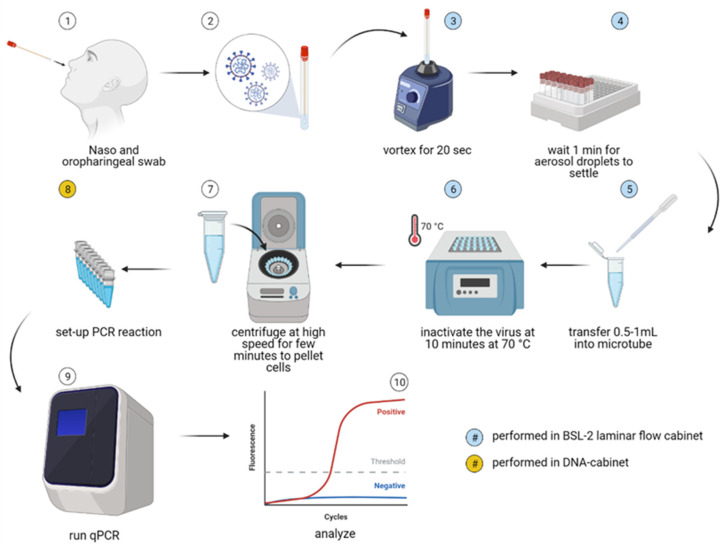
Scheme of the direct qPCR (dqPCR) method employed in this study. Nasopharyngeal and oropharyngeal swabs (1) were immersed in 2 mL of MG water (2), and samples were stored at +4 °C for a maximum of 5 h until further analysis. Within a Class II biosafety cabinet, samples were then vortexed for 20 s at high speed (3) and then left to rest for at least 1 min to minimize aerosol spread (4). Between 0.5–1 mL of each sample was then transferred into a separate qPCR grade microtube with a disposable Pasteur pipette (5). Viral particles were then inactivated by heating the samples at 70 °C for 10 min, and cellular debris was pelleted by centrifuging the samples in a tabletop microcentrifuge for 5 min at 1000× *g*. Following centrifugation, a small 10 µL sample aliquot was used directly in a qPCR reaction (8–10). Steps (3–7) and steps (8–10) were performed in physically separated laboratories to reduce the risk of sample cross-contamination. The figure was created with BioRender.com.

Tubes with swab samples were first vortexed for 20 s at high speed in a grade 2 laminar-flow biosafety cabinet and then left on the work surface for a minimum of 1 min to allow aerosols to settle. RNA from UTM samples was then extracted using the NucleoSpin RNA Virus isolation kit (Macherey Nagel) from 140 µL of UTM and eluted in 50 µL of MG water, according to the manufacturer’s instructions. For swab samples in MG water, the RNA isolation step was omitted. Instead, 0.5–1 mL of each sample in MG water was transferred with disposable Pasteur pipettes into RNA and RNAse-free 1.5 mL conical microtubes. Following the transfer, samples for dqPCR were heated at 70 °C for 10 min in a thermo-block to inactivate the potentially present SARS-CoV-2 particles and release the genome from virions. Inactivated samples were centrifuged at high speed (>2000× *g*) for 5 min to pellet cellular debris and collect condensate from the lids and then stored at +4 °C until qPCR was performed (Figure 1).

Allplex SARS-CoV-2 Assay (Seegene, Seoul, Korea) was used for the detection of SARS-CoV-2 by RT-qPCR. Procedures for stndqPCR and dqPCR as well as RT-qPCR sample reaction are summarized in Table 1.

Premade reaction mix for the POCT-style modification of the dqPCR was prepared by combining the entire contents of the Allplex SARS-CoV-2 Assay kit components, 500 µL MOM, 500 µL EM8, and 100 µL IC. Ten-microliter aliquots of the premade reaction mix were distributed into qPCR-grade microtube-strips and stored at −20 °C until use. RT-qPCR was performed on the 7500 FAST (Applied Biosystems, Thermo Fischer Scientific, Waltham, Massachusetts, USA) or CFX96Dx (Bio-Rad, Hercules, California, USA) instruments, according to the manufacturer’s instructions. On the CFX96Dx, the detection threshold was set automatically, and following export from Bio-Rad CFX Manager software, Cq values were determined by the Seegene Viewer application, according to the manufacturer’s instructions. On 7500 FAST, Cq was determined by the personnel after manually setting the detection threshold.

### 2.3. Generation of RT-qPCR Titration Curves

SARS-CoV-2 isolate 297/20 Zagreb was derived from an RT-qPCR-positive nasopharyngeal swab in Zagreb, Croatia, which was propagated four times in Vero E6 cells. The virus was sub-cultivated one more time by infecting Vero E6 cells at an MOI of 0.001 to obtain a working virus stock. Vero E6 cells were maintained in a 5% CO_2_ environment at 37 °C in MEM (GIBCO, Thermo Fisher Scientific), supplemented with 10% FBS (PAN-Biotech, Aidenbach, Germany), penicillin (100 IU/mL), streptomycin (100 µg/mL), and L-glutamine (2 mM) (Capricorn Scientific, Ebsdorfergrund, Germany). Two days post-infection, supernatants from infected cells were centrifuged for 10 min at 1500× *g*, aliquoted in 10% FBS, and aliquots stored at −75 °C. The infectivity of viral preparation was determined to be 6.83 ± 0.15 CCID_50_/mL (*n* = 8). NGS sequencing of the viral genome revealed that the isolate belonged to the B1.1.1. lineage. The stock solution of SARS-CoV-2 RNA for generation of titration curves was isolated from 200 µL of supernatant using the Quick-RNA Viral isolation kit (Zymo Research) and eluted in 20 µL of MG water. We generated six consecutive 10-fold serial dilutions of stock viral RNA using either MG water or SARS-CoV-2-negative pooled sample as the dilution mediums. Three technical replicates of each prepared serial dilution were then analyzed in an RT-qPCR reaction (5 µL of sample, 1 µL of IC (internal control) amplicon, and 14 µL of Allplex SARS-CoV-2 Assay reaction mixture) on the Applied Biosystems 7500 FAST instrument. Obtained Cq values were plotted against the log-amount of the virus, and generation of linear regression curves, efficiency, and R^2^ value calculations were performed using Applied’s 7500 FAST analysis software. Graphs were plotted in GraphPad Prism 8.

### 2.4. Statistical Analysis

Agreement between stndPCR, dqPCR, and POC dqPCR was quantified using kappa statistics, as implemented in GraphPad’s free online tool (https://www.graphpad.com/quickcalcs/kappa1.cfm, accessed several times between May and December 2021.). The Wilcoxon matched-pairs signed-rank was used to compare Cq values between matching samples by using GraphPad Prism 8.

## 3. Results

### 3.1. Adaptation and Validation of the Allplex SARS-CoV-2 qPCR Assay into a Direct qPCR Assay

To investigate whether the Seegene Allplex SARS-CoV-2 assay could be adapted into a dqPCR assay for SARS-CoV-2 detection utilizing the protocol schematically depicted in Figure 1, we compared the performance of Seegene’s recommended assay procedure at the time (standard qPCR or stndqPCR) with the modified, direct qPCR (dqPCR) assay in which oropharyngeal swab samples were directly subjected to qPCR analysis without performing viral RNA isolation and purification. Of note, at the time, the Allplex SARS-CoV-2 assay had not yet been validated for use without RNA isolation. 

**Figure 2:**
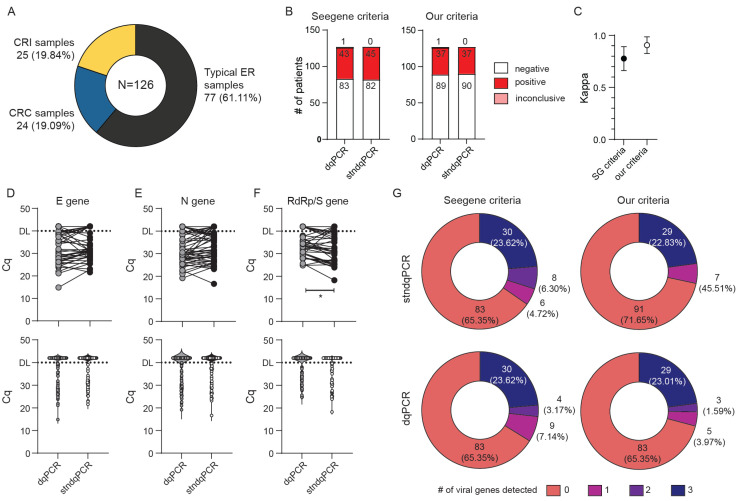
Sensitivity and specificity comparison of direct qPCR (dqPCR) and standard qPCR (stndqPCR). Only samples with valid control (IC) amplification were analyzed. (**A**) The patient structure included in the study: patients coming to the emergency department (ED) for initial COVID diagnostics (typical ER samples), convalescent patients (CRI) with confirmed infection with SARS-CoV-2 at least 2 weeks prior to this analysis, or patients hospitalized in the COVID respiratory center (CRC) of CHC Rijeka. (**B**) Diagnoses obtained by employing protocol recommended by the manufacturer with RNA isolation (stndqPCR) or direct qPCR (dqPCR) and utilizing criteria recommended by the manufacturer (Seegene criteria) or our criteria. The number of diagnoses per each protocol and criteria is shown inside bars. (**C**) Interrater agreement between diagnoses obtained with stndqPCR and dqPCR using kappa analysis utilizing two criteria for result interpretation. (**D**–**F**) Cycles of quantitation (Cq) for E gene (**D**), N gene (E), and RdRp/S gene (**F**) between stndqPCR and dqPCR. Upper panels depict samples from the same patient connected with the line. The bottom panels show violin plots of Cq values for all samples in the study. DL denotes detection limit in samples; above DL, no amplification was detected. Wilcoxon matched-pairs signed-rank test was used to determine whether Cq values differed significantly between groups, and statistical significance (*p* < 0.05) is labeled with *. (**G**) Pie charts showing the number of viral genes detected per test in two protocols and employing the two criteria for interpretation of the results. The number of patients with individual findings and corresponding percentage in the study are shown within each pie slice.

We recruited a total of 126 subjects for this comparison (Figure 2A), and the majority (77) were representative of the populations we expected the ED to analyze on a routine basis, such as patients arriving at the ED with COVID-19-like symptoms, patients requiring a SARS-CoV-2 test for hospital admission, or CHC Rijeka personnel working with confirmed or suspected COVID-19 patients. Furthermore, to account for situations in which samples could contain an increased amount of mucus and PCR inhibitors, we recruited another 24 critical-care patients (COVID respiratory center samples, CRC samples) diagnosed with severe COVID-19. Finally, to account for samples that could contain very low amounts of SARS-CoV-2 particles, we recruited an additional 25 convalescent patients that were tested positive for COVID-19 at least 2 weeks prior to sample collection. Following patient recruitment, we collected two swabs (samples) from each patient. Oropharyngeal and nasopharyngeal swabs were placed together into a universal transport medium (UTM), and each sample was analyzed by stndqPCR procedure, which included the RNA isolation and purification step, as recommended by the manufacturer. The second, oropharyngeal swab was placed into MG water and analyzed using the dqPCR procedure, which, as described in Figure 1 and Materials and Methods, bypasses the time-consuming RNA extraction step. We opted for the MG water as the preferred medium for storing swabs used for dqPCR since the UTM contains salts that could inhibit PCR if added directly into the reaction mixture [15]. Oro- and nasopharyngeal swabbing was performed by the experienced medical staff of CHC Rijeka and then processed at the University of Rijeka, Faculty of Medicine, Center for Proteomics, by researchers with extensive expertise in and knowledge of qPCR and RNA isolation, purification, and handling. Analysis of the obtained stndqPCR and dqPCR results and their stratification into SARS-CoV-2 positive, SARS-CoV-2 negative, or inconclusive category were performed utilizing two sets of criteria: criteria suggested by the manufacturer (Seegene, (SG) criteria) and our more stringent criteria (Table 2). Our rationale for implementing additional criteria was based on two concerns: (1) samples with very high Cq values contain a very low amount of viral genomes from the swab, which are likely not infectious [23,24,25] or (2) high Cq values, especially if only one virus-specific amplicon was detected, could be a consequence of cross-contamination in the lab during sample preparation [26]. In either case, false positive finding could result in non-COVID-19 patients being housed together with COVID-19 positive patients. Alternatively, no longer infectious, convalescent COVID-19 patients could be retained in hospital isolation, taking up space.

As shown in Figure 2B,C, the numbers of positive, negative, or inconclusive assay results obtained with dqPCR were consistently in strong agreement with those obtained with stndqPCR, while the agreement between the stndqPCR and dqPCR was even more pronounced when our criteria were used to stratify obtained results (κ = 0.77 for Seegene, and κ = 0.906 for our criteria). We did not observe a loss of sensitivity for E and N gene amplicons, while there was a statistically significant difference between the Cq values for RdRp/S amplicons between stndqPCR and dqPCR (Figure 2D–F). Violin plots of similar shapes were obtained when plotting Cq values for each virus-specific amplicon analyzed by the two methods (Figure 2D–F bottom panels). Moreover, both methods detected a comparable number of genes when identical criteria were used for interpreting results (Figure 2G). A total of five samples, all collected from intensive care patients undergoing treatment in the COVID respiratory center (CRC samples), gave invalid results when analyzed by dqPCR even though they were successfully analyzed using the stndqPCR procedure. Such an outcome was not unexpected since patients that require hospital care due to COVID-19 often have pneumonia, and their samples tend to contain high mucus content, which can negatively interfere with qPCR. The results from these five samples were excluded from Figure 2 and accompanying analyses.

**Figure 3:**
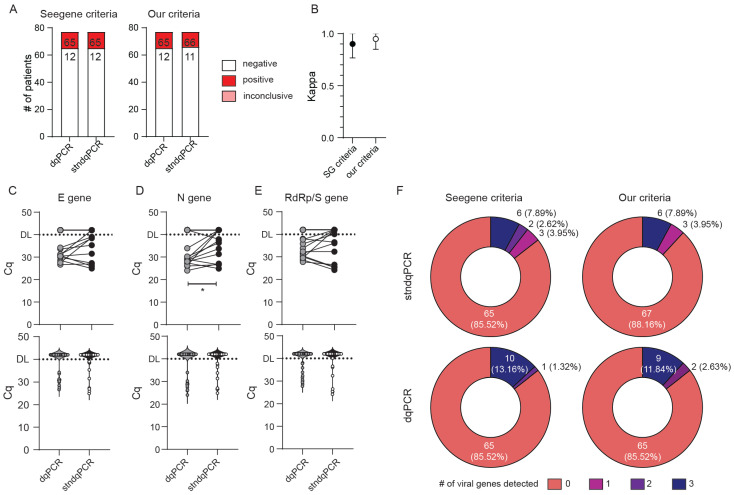
Sensitivity and specificity comparison of dqPCR and standard qPCR on typical ER samples. Only samples with valid internal (IC) gene amplification were analyzed. (**A**) Diagnoses obtained using a standard protocol with RNA isolation (stndqPCR) or direct qPCR (dqPCR) utilizing criteria recommended by the manufacturer (Seegene criteria) or our criteria. The number of diagnoses per each protocol and criteria is shown inside bars. (**B**) Interrater agreement between diagnoses obtained using stndqPCR and dqPCR and utilizing two criteria for result interpretation. (**C**–**E**) Cycles of quantitation (Cq) for E gene (**C**), N gene (**D**), and RdRp/S gene (**E**) between stndqPCR and dqPCR. The upper panels depict samples from the same patient connected with the line. The bottom panels show violin plots of Cq values for all samples in the study. DL denotes detection limit in samples; above DL, no amplification was detected. Wilcoxon matched-pairs signed-rank test was used to determine whether Cq values differed significantly between groups, and statistical significance (*p* < 0.05) is labeled with *. (**F**) Pie charts showing the number of viral genes detected per test in two protocols and employing the two criteria for interpretation of the results. The number of patients with individual findings and the percentage in the study are shown within each pie slice.

Since our goal was to design a test that could be utilized in the ED and similar near-patient settings, we performed identical analyses excluding CRI and CRC subjects. An even greater agreement between the two methods was obtained when only typical ED samples were analyzed (Figure 3) (κ = 0.901 (95% CI range 0.767–1.000) vs. κ = 0.949 (95% CI range 0.850–1.000) for Seegene vs. our criteria, respectively). As in the previous analysis, dqPCR was not associated with any loss in sensitivity of detection (Figure 3B–F). Indeed, we detected statistically significant lower Cq values for N gene in dqPCR compared with stndPCR (Figure 3D), which contributed to a greater number of viral genes detected in positive patients by dqPCR in comparison with stndPCR, regardless of the criteria used (Figure 3F). We thus concluded that the Allplex SARS-CoV-2 assay could be adapted to direct PCR without sensitivity and specificity loss. 

### 3.2. Adaptation of dqPCR into a Point-of-Care Type dqPCR for SARS-CoV-2 Detection in a Clinical Setting

Having shown that detection of SARS-CoV-2 by dqPCR does not suffer from loss of sensitivity or specificity, or from an unacceptable increase in the number of invalid samples, we set out to further optimize the dqPCR assay to more closely resemble the POC tests. To do so, we premixed all the reagents (EM8 and MOM containing Taq DNA polymerase, reverse transcriptase, buffer, dNTPs, primers, and probes) into a single premade reagent mix, dispensed 10 µL aliquots of the obtained reagent mix into qPCR-ready PCR strip tubes, and stored the aliquots at −20 °C. The suitability of the premade reagent mix for SARS-CoV-2 detection was then assessed by performing a qPCR assay on samples containing positive control RNAs included in the Allplex SARS-CoV-2 kit and either premade or freshly made reagent mixtures. We did not detect any differences between Cq values of any PCR products obtained using freshly made or premixed reagents (data not shown), regardless of whether water was included or excluded from the premixes. Namely, the standard qPCR mixture for Seegene’s Allplex SARS-CoV-2 assay contained 5 µL of MOM, 5 µL EM8, 5 µL water, and 5 µL of the sample. However, to further simplify the assay procedure and avoid pipetting errors associated with small volumes, we decided to exclude water from the premixes and instruct the ED personnel to add 10 uL of the heat-inactivated sample directly into premade reagent mixes. Positive and negative qPCR controls were also premade, dispensed into qPCR-ready PCR tubes, and stored at −20 °C. ED personnel were instructed to prepare samples as shown in Figure 1 and withdraw the required number of premade PCR strips from the −20 °C refrigerator immediately before running dqPCR. This modified dqPCR procedure, in which premade reagent mixes were used for the detection of SARS-CoV-2, was named POC-dqPCR.

**Figure 4:**
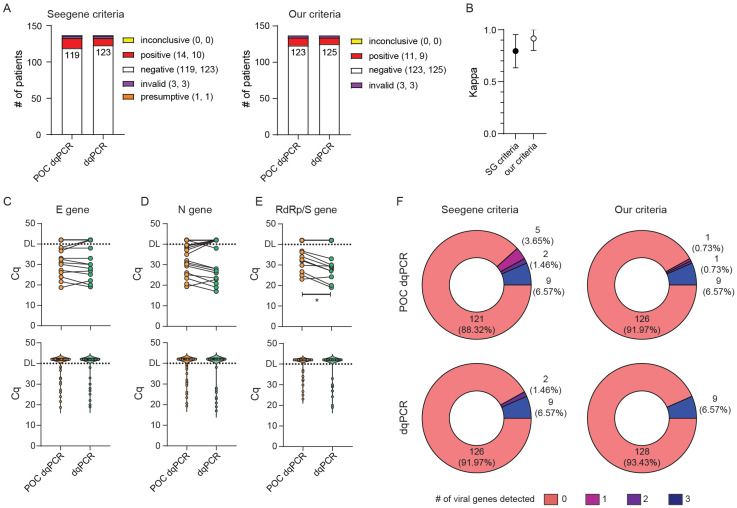
Application and validation of point-of-care (POC)-style dqPCR (POC dqPCR) in the ED CHC Rijeka. (**A**–**E**) Same samples from ED patients were analyzed in parallel utilizing POC dqPCR within the ED and dqPCR at the Center for Proteomics. (**A**) Diagnoses obtained by POC-dqPCR vs. dqPCR utilizing criteria recommended by the manufacturer (Seegene criteria) or our criteria. The number of diagnoses per protocol and criteria is shown in parentheses, where the first number is for POC dqPCR and the second for dqPCR. (**B**) Interrater agreement between diagnoses obtained using POC dqPCR and dqPCR utilizing two sets of criteria for result interpretation. (**C**–**E**) Cycles of quantitation (Cq) for E gene (**C**), N gene (**D**), and RdRp/S gene (**E**) between POC dqPCR and dqPCR. The upper panels depict samples from the same patient connected with the line. The bottom panels show violin plots of Cq values for all samples in the study. DL denotes detection limit in samples; above DL, no amplification was detected. Wilcoxon matched-pairs signed-rank test was used to determine whether Cq values differed significantly between groups, and statistical significance (*p* < 0.05) is labeled with *. (**F**) Pie charts showing the number of viral genes detected per test in two protocols and employing the two sets of criteria for result interpretation. The number of patients with individual findings and the percentage in the study are shown within each pie slice.

As a control for POC-dqPCR reliability, the same swab solutions were reanalyzed by an independent team of researchers at the Center for Proteomics who used freshly made PCR reaction mixes. Of note, the two teams used different qPCR instruments, with Bio-Rad’s CFX96Dx being used at the CHC ED and Applied Biosystem’s 7500 FAST at the Center for Proteomics. This fact might account for certain slight differences in the reported Cq values for individual genes since different systems utilize different strategies for setting quantification thresholds. In total, we evaluated 137 samples from patients and CHC employees undergoing routine SARS-CoV-2 testing and obtained Cq values were again evaluated according to the two sets of criteria shown in Table 2. As can be seen in Figure 4, the POC-dqPCR performed by medical personnel with minimal experience and training in qPCR yielded results that compared very favorably to the results of dqPCR performed by experienced researchers, independently from criteria used for stratification of results. Of all analyzed samples, three were invalid (no internal control amplification) and were thus excluded from further analyses. Applying our more stringent criteria also resulted in a better agreement between POC-dqPCR and dqPCR results (κ = 0.795 (95% CI range 0.954–0.634 for Seegene criteria vs. κ = 0.917 (95% CI range 0.802–1.000) for our criteria). Nevertheless, both sets of criteria resulted in a high degree of agreement between the POC-dqPCR and dqPCR. Furthermore, we observed no difference between Cq values for E and N amplicons and only a small change in Cq values for RdRp/S (Figure 4C–E, upper panels). Violin plot profiles were nearly identical (Figure 4C–E, lower panels), as well as the number of detected genes in the dataset (Figure 4F). We thus concluded that the POC-dqPCR, a modified dqPCR procedure in which premade reagent mixes are used, can be reliably utilized to detect SARS-CoV-2 within the ED or similar near-patient settings by personnel without prior experience in molecular diagnostics.

### 3.3. Validation of dqPCR Procedure Using Standardized Samples and Application to Pooled Sample Analyses

**Figure 5:**
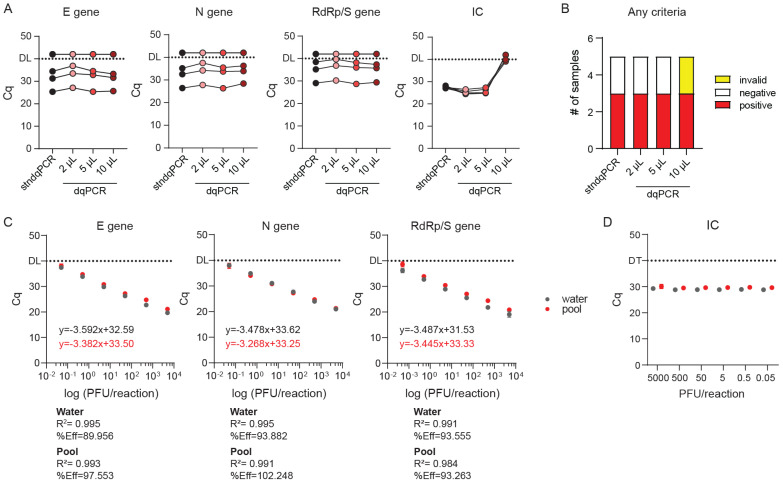
Sensitivity of dqPCR procedure in standardized samples (**A**,**B**) and applicability to pooled testing ©. (**A**) Validation of dqPCR method on standardized WHO-approved validation samples. Cq values of individual detected amplicons for stndPCR vs. dqPCR at different amounts of sample per reaction. (**B**) Diagnoses based on the results shown in A. (**C**) Titration curve using SARS-CoV-2 genome diluted in water or a negative pool sample and analyzed using dqPCR protocol. (**D**) Internal control (IC) amplification in reactions with different reference RNA concentrations diluted in either water or negative pool.

We initially utilized high-purity MG water as a medium for oro- and nasopharyngeal swab resuspension to avoid potential inhibition of the dqPCR due to increased salt content and inhibitors in universal transport medium. However, patient swabs from other CHC departments would arrive at the ED in UTM or some other saline solution rather than MG water. In addition, recent reports suggested that the dqPCR procedure could be performed successfully on UTM samples using the Seegene Allpex SARS-CoV-2 Assay [16,27]. Therefore, we decided to investigate whether dqPCR could be applied to UTM/saline solutions in our settings. In addition, to further validate the suitability of dqPCR for SARS-CoV-2 detection, we took advantage of the World Health Organization’s (WHO) Laboratory Proficiency Testing initiative organized in December 2020. We applied to the initiative and obtained a total of five gamma-irradiated samples from WHO. All samples contained PBS and 0.5% gelatin; three contained high, medium, and low amounts of SARS-CoV-2 genome, respectively. The fourth sample served as a negative control, and the fifth sample contained coronavirus HCoV-OC43 material to test whether the implemented assay could specifically detect SARS-CoV-2 and not some other viruses from the coronavirus family. We analyzed these samples with both stndqPCR and dqPCR using three different volumes of the template: 2, 5, and 10 µL (Figure 5A,B). Based on the obtained Cq values for E, N, and RdRp/S amplicons, dqPCR utilizing 5 and 10 µL of the sample was equally as sensitive as stndqPCR, while dqPCR in which 2 µL of the sample were used as a template was slightly less sensitive. Inhibition was observed only in internal control amplicons when 10 µL of the samples were used as a template, consistent with inhibition due to increased salt concentration. Most importantly, utilizing dqPCR, we consistently identified all five WHO samples correctly, regardless of the sample volume used as a template. These results indicate no loss of sensitivity or specificity when using dqPCR for SARS-CoV-2 detection, even if samples are delivered in saline rather than high-purity MG water (Figure 5A).

Next, we used RNA from in vitro grown SARS-CoV-2 clinical isolate 297/20 Zagreb of known titer to generate titration curves and determine the sensitivity of our assay. Since purified RNA diluted in water is not representative of a typical sample for dqPCR assay, and since we wanted to evaluate the suitability of dqPCR to pooled samples from multiple patients, we additionally diluted the reference RNA in a pooled sample containing oropharyngeal swabs from five CHC Rijeka employees placed in 2 mL of MG water, previously determined to be negative for SARS-CoV-2. If components of saliva and swabs could inhibit dqPCR reaction, we would observe it in titration curves. As shown in Figure 5C, the Cq values for all virus-specific amplicons were almost identical regardless of whether reference virus RNA was diluted in molecular grade water or SARS-CoV-2-negative pool sample. In addition, none of the samples caused noticeable PCR inhibition at any concentrations used. We could also see no difference in the Cq values of internal control between samples containing pure virus RNA diluted in MG water or a negative pool either between the same dilutions or across different dilutions (Figure 5D). We conclude that dqPCR can be applied even if samples are delivered in UTM/saline; however, in that case, instead of 10 µL we recommend using 5 µL of the sample as a template. We thus conclude that dqPCR can effectively and reliably be applied to detect SARS-CoV-2 in pooled samples (multiple swabs in a single volume of MG water).

### 3.4. Application of POC-dqPCR within the ED Department—A 4-Month Analysis

**Figure 6:**
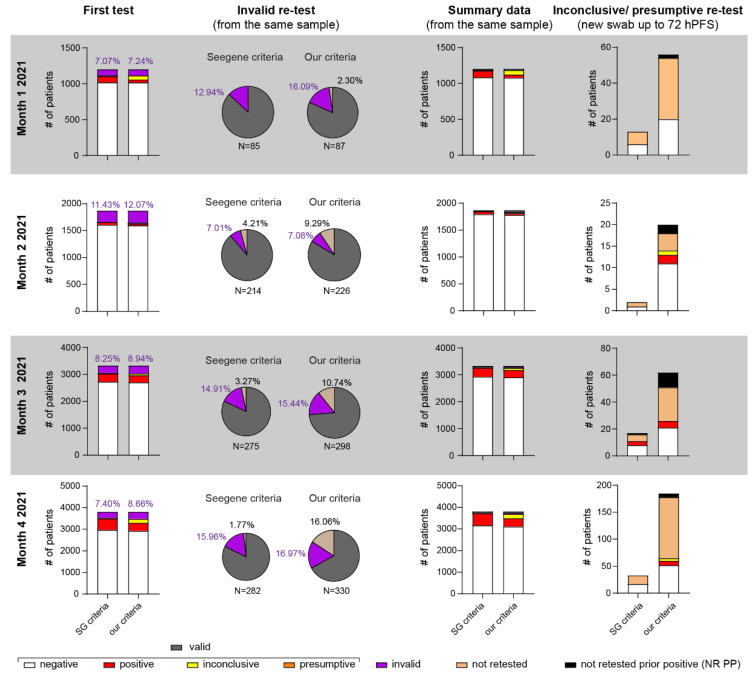
Application of POC-dqPCR in the ED setting—4 month analysis. First column shows distribution of results following first test. All invalid samples (no amplification in IC (SG criteria) or amplification ≤37 in our criteria) were retested from the same sample (second column). Monthly summary data (third column) shows data after invalid retest and added to the valid results of the first test. Wherever possible, presumptive (Seegene criteria) or inconclusive (our criteria) patients were re-tested by taking a new sample 24–72 h post first sampling (hPFS) unless these patients had a known prior COVID-19 infection (NR PP = not retested, known prior positive).

Having validated the dqPCR and POC-dqPCR methods, we next employed POC-dqPCR within the ED of CHC Rijeka. In 4 months, over 10,000 samples were analyzed, primarily by medical doctors in the ED with little prior experience with qPCR. As shown in Figure 6, less than 10% of samples were invalid due to failure of IC amplification in the first test in all months analyzed except in month 2. A great majority were successfully reanalyzed from the same swab sample in a second try, bringing the test success rate very close to 99% (Table 3). 

To account for the possibility that some of the patients diagnosed as presumptive by Seegene’s or inconclusive by our criteria (Figure 6, last column and Table 3) were in fact at the beginning of the infection, whenever possible, we attempted to acquire a new sample within 24–72 h after the first sample (hPFS). This time frame was chosen to minimize the possibility of a new exposure to virus that could confound the results. Again, most such samples were true negative, under both our and Seegene criteria (Table 4). Inconclusive or presumptive samples from patients with known SARS-CoV-2 infection (positive test up to 1 month prior) were not retested, as long-term low-level viral RNA detection in some COVID-19 positive patients for several weeks is a well-described phenomenon in the literature [4,28,29].

In total, 15 patients (4.16% of all inconclusive patients) were retested as positive by taking a new swab within 72 h PFS in a 4-month period, and the results of their tests and clinical data available at the time of testing are shown in Table 5. Of these 15 patients, five (33%) were known COVID-19 positive patients that required retesting for various reasons. four (26.67%) had COVID-19-like symptoms and only four (26.67%) were newly diagnosed, completely asymptomatic patients, mostly tested as part of surveillance testing (as hospital employees or patients requiring hospital admission).

In total, only 4 out of 361 (1.1%) inconclusive patients retested as positive and had no symptoms or clinical or epidemiological reasons that would suggest COVID-19 infection and could have thus been designated as false negatives. In contrast, by utilizing less stringent Seegene criteria, most of these patients might have been misdiagnosed as false positives.

We concluded that our POC-dqPCR is a reliable method and can easily be implemented in EDs or other near-patient settings. We further concluded that diagnostic criteria for high Cq values, near the test’s detection limit, should be evaluated based on available clinical data whenever possible, considering the current incidence of SARS-CoV-2 infection in the population.

## 4. Discussion

In this study, we investigated the adaptability of a standard RT-qPCR IVD test by Seegene to a point-of-care type test and applicability of the modified test for SARS-CoV-2 diagnostics within the Emergency department of Clinical hospital Rijeka. To simplify the procedure and increase testing speed and capacity, we decided to omit the viral RNA isolation step. Two primary purposes of RNA isolation before qPCR are virus inactivation, concentrating RNA, and removing possible PCR inhibitors (e.g., hemoglobin) from the sample. We inactivated the virus by heating aliquots of swabs at 70 °C for 10 min, which was previously demonstrated to be sufficient for SARS-CoV-2 inactivation [13]. In recent years, many commercially available products of reverse transcriptases (RT) and DNA polymerases have developed robust enzymes that are resilient to common RT and PCR inhibitors and can handle complex samples without the need for nucleic acid purification. We validated the feasibility and sensitivity of such a direct qPCR approach on 126 samples (Figure 2). Unlike some other reports [15], we did not find that dqPCR resulted in diminished sensitivity, probably because we chose MG pure water instead of UTM as our swab solution. Moreover, because our swab solution was MG water, we could use double the amount of sample and omit water from the reaction mixture, simplifying the procedure. This, along with faster processing time and robust enzymes present in the reaction mixture, could explain why dqPCR and stndqPCR were equally sensitive.

We then tested whether all components of the RT-qPCR reaction could be premixed, dispensed into qPCR tubes, and frozen again to be used as a POCT-style test, as was already described for the PrimeDirect™ Probe RT-qPCR Mix (TaKaRa) kit [12]. A significant modification in our protocol was the implementation of such dqPCR with premixed reagents into the regular ED setting, where the assay was performed by medical doctors with very little or no prior experience with qPCR, laboratory work, or molecular diagnostic procedures in general. Although numerous POCTs are available for detecting SARS-CoV-2, they are expensive, usually require specialized instrumentation, and can process only one or a small number of samples in parallel [3]. Furthermore, unlike antigenic tests and LAMPs, our protocol does not sacrifice sensitivity for speed and simplicity and provides users the freedom to choose any reagent and qPCR instrument. Indeed, we worked in parallel on the CFX96Dx (Bio-Rad) and 7500 FAST (Applied Biosystems) and observed concordant results (Figure 3, and not shown). 

In addition to our validation, we tested POC-dqPCR using WHO’s Laboratory Proficiency Testing initiative samples (December 2020). Even though these samples were prepared in saline, POC-dqPCR correctly identified all SARS-CoV-2 positive and negative samples with sensitivity comparable to qPCR performed using isolated and purified RNA as a template. We observed slight inhibition of internal control sample amplification when we added 10 µL of WHO samples directly into the POC-dqPCR reaction, likely because this higher sample volume significantly increased the concentration of salts in the reaction. Still, even with the inhibition of IC amplicons, POC-dqPCR still correctly identified all SARS-CoV-2 positive samples. The absence of inhibition of SARS-CoV-2 specific targets could be an intentional design feature of the test, where IC primer sets and reaction are intentionally designed as suboptimal so that it will not compete with amplification of SARS-CoV-2 positive amplicons that are expected to be present at lower concentrations in the sample. 

We also evaluated the sensitivity of POC-dqPCR using samples “spiked-in” with RNA from clinical SARS-CoV-2 isolate of known infectivity (TCID_50_), diluted both in pure MG water and pooled sample. Virus RNA was detectable even at the highest dilution, corresponding to 0.05 PFU/reaction, which is in line with previously published data reporting 3–5 orders of magnitude difference between RNA copy number and titer determined by TCID_50_ [30]. Furthermore, there was no difference in the titration curve for any amplicon between standard viral RNA diluted in MG water or in previously tested negative pooled sample, indicating that POC-dqPCR could also be applied to pooled analyses (manuscript in preparation). 

Finally, following implementation of POC-dqPCR in the near-patient setting of the ED, we performed a 4-month analysis of the obtained diagnostic results utilizing Seegene’s and our own modified criteria. As can be seen in Figure 6, we observed a failure of IC to amplify in around 10% of samples, rendering them invalid. In 80% of these invalid samples, IC could be detected after retest from the same sample, resulting in less than 1% of failed tests that required re-sampling from the patient. We hypothesize that the likely reasons for these failed tests are pipetting errors during PCR setup. 

There has been much discussion on the threshold setting for determination of COVID-19 positive and negative results. Set the Cq threshold too high, and one risks false positives, resulting in uninfected patients being housed with COVID-19 positive patients or retaining no-longer-infectious convalescent patients in isolation and straining hospital resources. Setting the Cq threshold too low risks false negatives. We thus tested an additional set of more stringent criteria requiring a Cq of 37 for IC for a sample to be considered valid instead of Seegene’s recommended 40. Our criteria also required at least two virus-specific amplicons detected at Cq lower than 38 to consider a sample positive (Table 2). A similar strategy has already been tested for Seegene’s Allplex SARS-CoV-2 assay but on fewer patients [31]. Whenever possible, we retested patients that had been classified as “COVID-19 presumptive” (SG criteria) or “inconclusive” (our criteria) by taking a new swab sample up to 72 h post first swab. We did not resample patients who tested positive for COVID-19 up to 1 month prior to the new test. As shown in Figure 6, last column, and Table 3, a great majority of “presumptive” and “inconclusive” patients were retested as negative. Of those retested as positive, only four were newly diagnosed patients with no prior history of COVID-19 positivity or contact with COVID-19 patients. The number of these potential “false negatives” increased with the increased incidence of COVID-19 positives in the population regardless of the criteria utilized. These results further underscore the importance of interpreting results with high Cq values in the context of clinical and epidemiological history.

In conclusion, we adapted Seegene’s Allplex SARS-CoV-2 assay into a POC-like direct qPCR assay that can efficiently and reliably be performed by medical staff within the Emergency department of the CHC Rijeka, and elsewhere.

## Figures and Tables

**Table 1:** Summary of the dqPCR and stndPCR Methods.

Procedure	stndqPCR	dqPCR
RNA isolation	On-column RNA isolation from 140 µL of UTM with 10 µL of IC added prior to isolation. Eluted into 50 µL of MG water.	None
Virus inactivation	None ^1^	Aliquot of cca 1 mL heated at 70 °C for 10 min
qPCR reaction	5 µL purified RNA sample 5 µL MOM 5 µL EM8 5 µL MG water	10 µL heat inactivated sample 5 µL MOM 5 µL EM8 1 µL IC

^1^ No additional inactivation was necessary since the process of RNA isolation includes destruction of the virions and therefore inactivates the virus.

**Table 2:** Criteria for interpretation of qPCR results. NA = not amplified.

Outcome	Seegene (SG) Criteria	Our Criteria
SARS-CoV-2 positive	Amplification of any SARS-CoV-2 specific amplicon (N, RdRp/S plus/minus E gen) at Cq < 40.	Amplification of any SARS-CoV-2 specific amplicon (N, RdRp/S) at Cq < 37 or two viral amplicons at Cq < 38.
SARS-CoV-2 negative	No amplification or Cq ≥ 40 for all viral amplicons. Cq for IC < 40.	No amplification or amplification of virus amplicons at Cq ≥ 38 or amplification of any single virus amplicon at Cq ≥ 37. Cq for IC < 37. The analysis is repeated if Cq for IC is ≥ 37.
Presumptive	Amplification of just E gene at Cq < 40.	Does not exist in our criteria.
Inconclusive	Does not exist in Seegene criteria.	Amplification of any SARS-CoV-2 specific amplicon—one (N, RdRp/S) at Cq ≥ 37 or two viral amplicons (E and N, E and RdRp/S, N and RdRp/S) at Cq ≥ 38.
Invalid	No amplification of any amplicons.	No amplification of any amplicons and IC ≥ 37.

**Table 3:** Summary data from 4-month analysis of POC-dqPCR after one retest of invalid samples graphically shown in Figure 6. Percentage and actual numbers are shown (in parentheses).

Month	Criteria	Negative	Positive	Inconclusive	Presumptive	Invalid	Not Retested	Total
Month 1	SG	90.35% (1086)	7.65% (92)	NA	1.08% (13)	0.92% (11)	0.00% (0)	1202
	OC	89.93% (1081)	3.49% (42)	5.24% (63)	NA	1.16% (14)	0.17% (2)	
Month 2	SG	95.84% (1795)	2.78% (52)	NA	0.11% (2)	0.80% (15)	0.48% (9)	1873
	OC	95.14% (1782)	1.76% (33)	1.12% (21)	NA	0.85% (16)	1.12% (21)	
Month 3	SG	87.97% (2931)	9.93% (331)	NA	0.60% (20)	1.23% (41)	0.27% (9)	3332
	OC	87.15% (2904)	8.37% (279)	2.13% (71)	NA	2.13% (46)	0.00% (0)	
Month 4	SG	83.20% (3169)	14.49% (552)	NA	1.00% (38)	1.18% (45)	0.13% (5)	3809
	OC	81.67% (3111)	10.06% (383)	5.41% (206)	NA	1.47% (56)	1.39% (53)	

**Table 4:** Retest of inconclusive or presumptive results graphically depicted in Figure 6. Percentage and actual numbers are shown (in parentheses). Patients were retested by taking another swab up to 72 h post first swab. NR PP = not retested, known prior positive. NA = not applicable.

Month	Criteria	Negative	Positive	Inconclusive	Presumptive	Invalid	Not Retested	NR PP	Total
Month 1	SG	46.15% (6)	0.00% (0)	NA	0.00% (0)	0.00% (0)	53.84% (7)	0.00% (0)	13
	OC	35.71 (20)	0.00%	0.00% (0)	NA	0.00% (0)	60.71% (34)	3.57% (2)	56
Month 2	SG	50.00% (1)	0.00% (0)	NA	0.00% (0)	0.00% (0)	50.00% (1)	0.00 (0)	2
	OC	55.00% (11)	10.00% (2)	5.00% (1)	NA	0.00% (0)	20.00% (4)	10% (2)	20
Month 3	SG	47.06% (8)	17.65% (3)	NA	0.00% (0)	0.00% (0)	29.41% (5)	5.88% (1)	17
	OC	33.87% (21)	8.06% (5)	0.00% (0)	NA	0.00% (0)	40.32% (25)	17.74% (11)	62
Month 4	SG	51.52% (17)	0.00% (0)	NA	0.00% (0)	0.00% (0)	48.48% (16)	0.00% (0)	33
	OC	28.11% (52)	4.32% (8)	2.70% (5)	NA	0.00% (0)	61.08% (113)	3.78% (7)	185

**Table 5:** Cq values from first test and retest on a new sample (first and second value, respectively) and available clinical data at the time of testing for patients with inconclusive test (our criteria) that retested as positive. Cq values from tests performed in a different laboratory were not included, as Cq values are not directly comparable between different tests.

Month	E (Cq)	N (Cq)	RdRp/S (Cq)	Available Clinical Data at Time of Testing
Month 2	39.87 30.27	39.40 29.80	NA 33.35	known COVID patient treated in critical care
	NA NA	39.72 Other lab	NA Other lab	newly diagnosed, with pneumonia
Month 3	34.13 23.48	NA 25.27	NA 33.03	newly diagnosed, mild upper respiratory symptoms
	38.2 Other lab	NA Other lab	NA Other lab	known COVID patient
	39.91 34.43	NA 33.67	NA NA	newly diagnosed, asymptomatic, surveillance testing
	39.53 Other lab	NA Other lab	NA Other lab	newly diagnosed, asymptomatic, dialysis patient
	39.04 Other lab	37.16 Other lab	38.9 Other lab	known COVID patient
Month 4	NA Other lab	39,93 Other lab	NA Other lab	known COVID patient
	NA Other lab	37.74 Other lab	NA Other lab	known COVID patient
	NA Other lab	39.54 Other lab	39.99 Other lab	newly diagnosed, surveillance testing
	NA NA Other lab	39.34 38.,51 Other lab	NA NA Other lab	newly diagnosed, surveillance testing
	NA 30.35	38.83 29.85	NA 36.53	newly diagnosed, treated for deep vein thrombosis, no respiratory symptoms
	NA 36,3	37,04 33,49	NA NA	newly diagnosed, critical-care patient, had respiratory symptoms for 7 days before first test
	NA 34.18	37.74 33.28	NA NA	newly diagnosed, asymptomatic
	NA Other lab	37.36 Other lab	NA Other lab	newly diagnosed, had respiratory symptoms for 14 days prior to testing

## Data Availability

Not applicable.
